# Supplementary figures and images for: pUL21 regulation of pUs3 kinase activity influences the nature of nuclear envelope deformation by the HSV-2 nuclear egress complex
Source: PLoS Pathog. 2021 Aug 23;17(8):e1009679. doi: 10.1371/journal.ppat.1009679 (PMC8412291; doi:10.1371/journal.ppat.1009679)

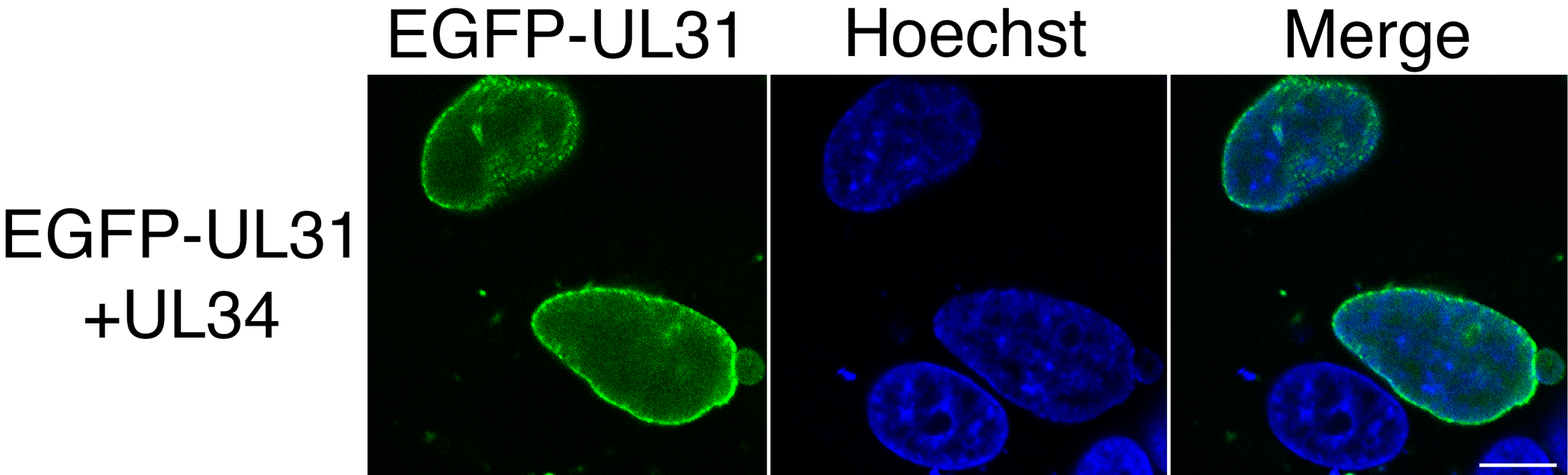

Supplement: S1 Fig — HeLa cells were co-transfected with the expression plasmids indicated on the left of the panel. At 18 hours post transfection, cells were fixed and nuclei were stained with Hoechst 33342. A representative image of cells with dim fluorescence indicative of modest NEC production is shown. Note that the distribution of the NEC differed from that of cells with bright fluorescence indicative of robust NEC production shown in the top row of images in Fig 4B. (TIF) [file ppat.1009679.s001.tif]

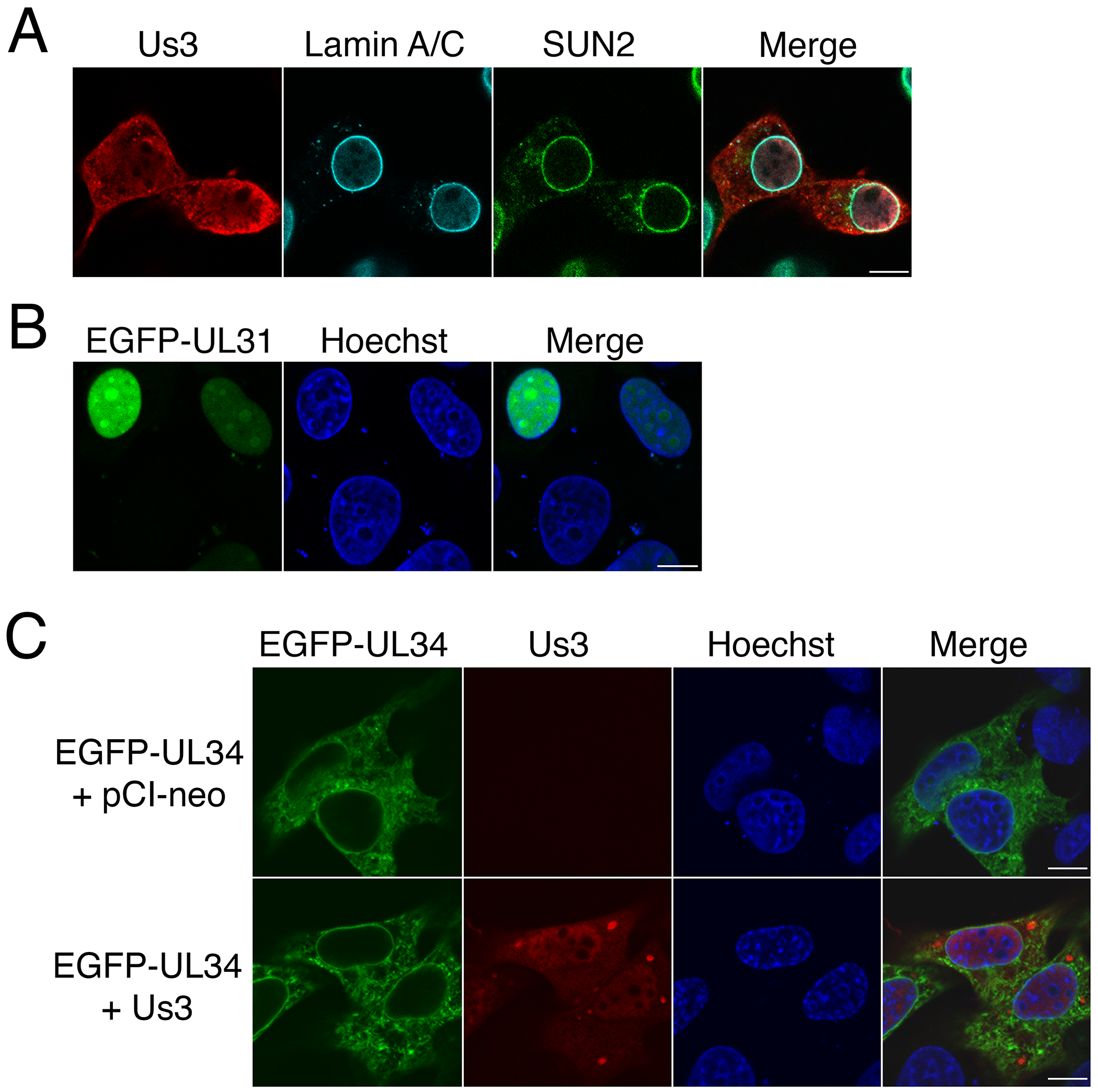

Supplement: S2 Fig — (A, B, C) HeLa cells were transfected with expression plasmid(s) and fixed at 18 hours post transfection. (A) Cells were transfected with a pUs3 expression plasmid and stained with primary antibodies specific for the protein indicated on the top of each image and Alexa Fluor-conjugated secondary antibodies. (B) Cells were transfected with EGFP-pUL31 expression plasmid. (C) Cells were co-transfected with the expression plasmids indicated on the left of each panel and stained with antibody specific for pUs3 and Alexa Fluor-conjugated secondary antibody. Nuclei in (A), (B) and (C) were stained with Hoechst 33342. Representative images are shown and all scale bars are 10 μm. Note that extravagations comparable those observed in cells with robust production of EGFP-pUL31, pUL34 and pUs3 (Fig 4B) were not observed in these control transfections. (TIF) [file ppat.1009679.s002.tif]

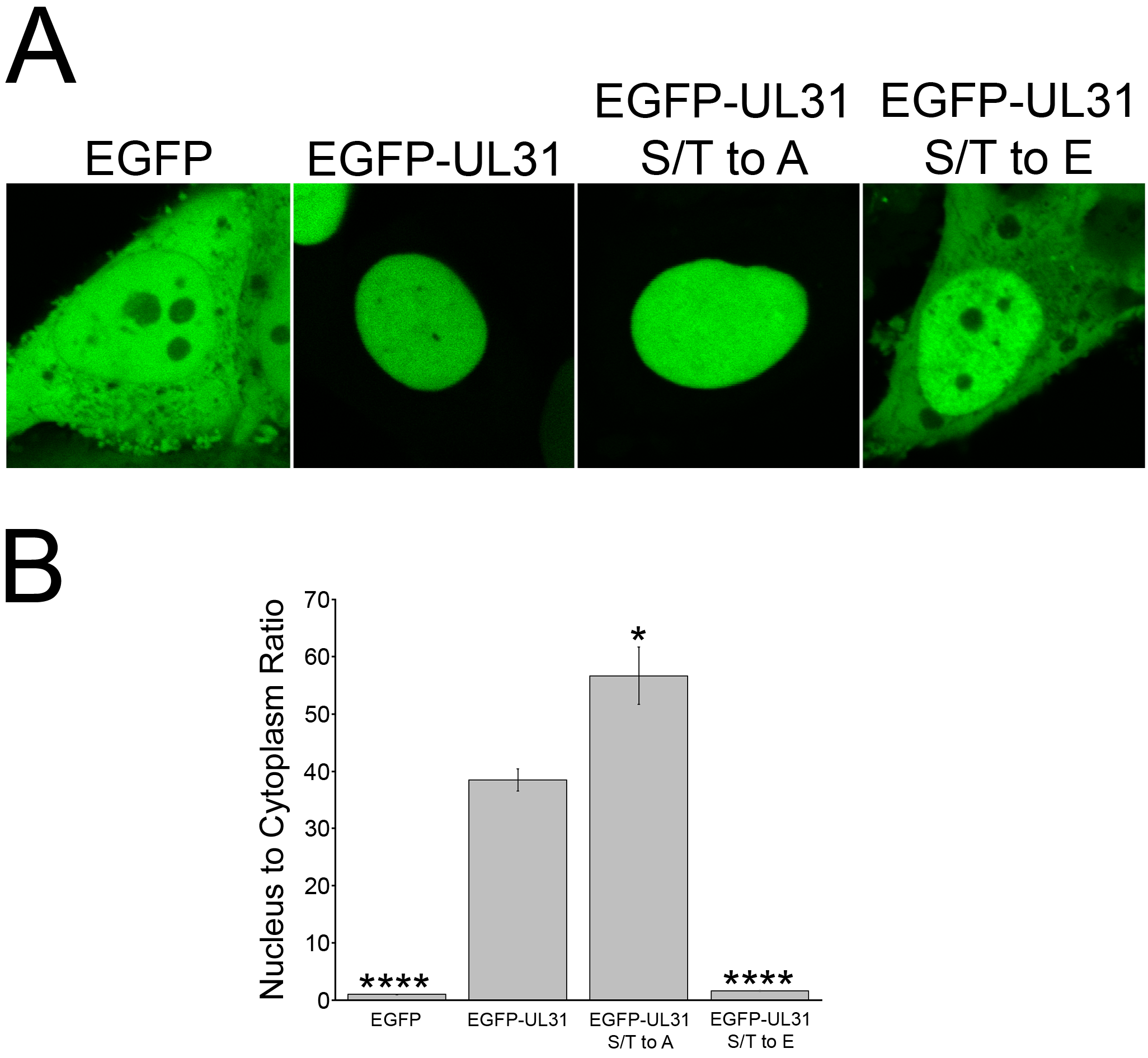

Supplement: S3 Fig — (A) HeLa cells were transfected with plasmids encoding the proteins indicated at the top of the micrographs. Images of EGFP fluorescence in live cells were collected by confocal microscopy. (B) Quantification of nucleus to cytoplasm ratios of EGFP fluorescence in living cells. Regions in the nuclei and cytoplasm of live cells were selected and the average fluorescence intensity within those regions measured using Fluoview software version 1.7.3.0. and the data were exported into Microsoft Excel for graphical presentation as described in Sherry et al. (26). EGFP n = 22, EGFP-pUL31 n = 18, EGFP-pUL31 S/T to A n = 8, EGFP-pUL31 S/T to E n = 7. Asterisks denote significant differences in the ratio of nuclear to cytoplasmic fluorescence in comparison to EGFP-pUL31 determined by Student’s T test; * = P value < 0.05, **** = P value < 0.0001. (TIF) [file ppat.1009679.s003.tif]
